# Supplementary material for: Hydrological control of river and seawater lithium isotopes
Source: Nat Commun. 2022 Jun 10;13:3359. doi: 10.1038/s41467-022-31076-y (PMC9187753; doi:10.1038/s41467-022-31076-y)
Supplement: Supplementary file 1 — Supplementary Information [file 41467_2022_31076_MOESM1_ESM.pdf]

Supplementary Information for

Hydrological control of river and seawater lithium isotopes

Fei Zhang[1,2*], Mathieu Dellinger[2,3], Robert G. Hilton[2,4], Jimin Yu[5,6], Mark B. Allen[7], Alexander L. Densmore[2], Hui Sun[1] & Zhangdong Jin[1,5,8*]

The Supplementary Information include:

1. Supplementary Note 1: Dataset of seasonal δ^7Li variations at diverse latitudes.
2. Supplementary Note 2: Variable seawater Li residence times.
3. Supplementary Note 3. Assessment of anthropogenic activities on seasonal Li variation.
4. Supplementary Figures 1 to 18.
5. Supplementary Table 1: Changes in seawater Li residence time in response to river influx.
6. Supplementary Table 2: Li contents and isotopes of Yangtze draining the two largest urban cities.

Supplementary Note 1. Dataset of seasonal δ^{7}Li variations at diverse latitudes

We have compiled available datasets to examine whether these seasonal patterns are worldwide. For the high-latitude Yenisei River[1] (with the largest annual Q_w draining into the Arctic Ocean), the monthly δ^{7}Li of river waters vary from 24.1‰ in low Q_w to 20.3‰ in high Q_w over the course of the hydrological cycle (Supplementary Fig. 6b).

In the mid-latitude regions, seasonal δ^{7}Li in a small forested granitic Strengbach catchment[2] (Vosges Mountains, France) vary from 16.9‰ in low Q_w to 12.4‰ in high Q_w (Supplementary Fig. 6d). The seasonal δ^{7}Li in basalt-dominated river basins are observed in Columbia River systems[3] for streams draining the west of the Cascades (wet conditions), where the seasonal difference in precipitation between wet and dry seasons is greatest (monthly precipitation differences are up to 10 times[3]). These streams have systematically higher δ^{7}Li during the dry season (Supplementary Fig. 5b). In contrast, in the areas with minor seasonal precipitation differences, the δ^{7}Li show minor seasonal variations (Supplementary Fig. 5b). The δ^{7}Li of river waters from the middle reaches of the Yellow River[4], draining the extensive Chinese Loess Plateau, are systematically lower in summer (wet season) than during the winter (Supplementary Fig. 6c). The weekly δ^{7}Li of the Yellow River waters varies from 16.9‰ to 21.5‰ during a whole year period[25], with the Q_w varying from 167 to 2401 m^3/s.

At low-latitudes, in rivers from the headwaters of the Ganges-Brahmaputra river system draining the Himalayas[5] and in the three largest tributaries (the Ganges, Brahmaputra and Meghna (G-B-M) Rivers) draining the downstream floodplain[6], the riverine δ^{7}Li are also higher in dry seasons and lower during the wet seasons (Fig. 2, Supplementary Fig. 5c-d). The δ^{7}Li in the headwaters vary from 13.7‰ to 25.9‰ in the dry season to from 11.2‰ to 19.4‰ in the wet season[5]. Similarly, the δ^{7}Li in the three main tributaries, as well as in the G-B-M mainstem after mixing of the Ganges, Brahmaputra and Meghna Rivers[6], vary from 23.8‰ to 30.5‰ in the dry season to from 19.1‰ to 26.5% in the wet season. Among them, the largest

seasonal variation (7.6‰) is observed in the Brahmaputra River sourced from the interior Tibetan Plateau (Fig. 2).

Across the equator in tropical rainforest regions, in the Congo River[7], Earth's second largest river system, the δ^7Li at the river mouth varies from 22.1‰ at low Q_w to 14.7‰ at high Q_w (Supplementary Fig. 6e). In the Amazon River, the δ^7Li of river waters also exhibited significant seasonal variations. The two main tributary, the Madeira River and the Rio Negro, along the Amazon mainstem all showed systematically higher δ^7Li at low Q_w but lower at high Q_w, even though they drain distinct geomorphic zones (Andes/floodplain versus Precambrian shield)[8].

The high consistency of these seasonal δ^7Li across latitudes suggests: 1) a common hydrology dependence of riverine Li isotopes; 2) in individual basins analyzed here, tributary mixing is not a major factor controlling their Li isotopic behaviors. In particular, the samples from the most upstream (headwaters of the Ganges), to the mouths of the 3 major tributaries (the Ganges, Brahmaputra and Meghna), and then to the G-B-M mouth all show systematically lower δ^7Li in wet relative to dry seasons. This is also consistent with observations in the Amazon river systems[8].

Supplementary Note 2. Variable seawater Li residence times

Modern river data (both seasonal and spatial) from the Arctic to the equator show that although increased runoff dilutes Li concentrations in river waters, more Li with lower δ^7Li is delivered into the ocean from high runoff rivers (Fig.3 and Supplementary Fig. 18). This may have important implications for understanding long-term evolution of Earth's surface processes on Li fluxes into the oceans. Given that our dataset encompasses a large range of vastly contrasting climate, vegetation (from the extremely cold dry Arctic to the warm wet equator rainforest), basin size (from small catchments to Earth's largest rivers), and distinct geomorphic

settings (Arctic permafrost, flat lowland shields, Rocky and Andean mountains, Loess Plateau, Pamir-Tibetan Plateaus, Himalayan floodplains, and tropical rainforests), we suggest that a decrease in long-term continental runoff would be associated with a decrease in Li flux to the oceans, with the effect of increasing Li residence time in the ocean and seawater δ^7Li.

We have employed a simple box model to quantitatively assess runoff effects on seawater Li residence time (τ). The mean τ is given by:

$$\tau = M / \sum(F_x)$$

where M is the inventory of dissolved Li in the ocean, and F_x represents the total input of Li fluxes to the ocean from hydrothermal (F_{HT}), subduction reflux (F_{Reflx}) and continental rivers (F_{Riv}). Assuming stable inputs of F_{HT} and F_{Reflx} as suggested by ref.[9], seawater Li residence time decreases with increasing F_{Riv}. Relative to the modern F_{Riv} input[9] to the ocean, halving this influx increases τ from 1.2 Ma to 1.4 Ma, while tripling the input decreases the τ to 0.7 Ma (Supplementary Table 1).

Supplementary Table 1. Changes in seawater Li residence time in response to river influx.

			Li-flux (10^9 mol yr^{-1})	Residence time (Ma)
Oceans-Li reservoir[9]		34*10^{16} mole		
Input to oceans	Input 1	Hydrothermal vents[9], F_{HT}	13	
	Input 2	Subduction reflux[9], F_{Reflx}	6	
	Input 3	River input-Scenario#1, F_{Riv}	5	1.4
		River input-Scenario#2 (modern)	10	1.2
		River input-Scenario#3, F_{Riv}	20	0.9
		River input-Scenario#4, F_{Riv}	30	0.7

As riverine Li flux changes, two processes can affect seawater δ^7Li. If continental riverine δ^7Li remains stable, a decrease in continental runoff would decrease F_{Riv} to the ocean, resulting in an increase in τ and thereby an increase in seawater δ^7Li[10]. At the same time, decreased runoff would deliver higher river water δ^7Li to the oceans (Fig.3 and Supplementary Fig. 18). Whatever the process, we expect to see an inverse covariation between continental hydrological

and seawater δ^7Li changes.

Supplementary Note 3. Assessment of anthropogenic activities on Li seasonal variation.

Recent studies show that anthropogenic input can increase riverine Li concentrations ([Li]) and decrease δ^7Li in the Han River, South Korea[11]. This raises a question whether anthropogenic activities may affect seasonal variations of δ^7Li in lowland rivers draining large urban areas with high population density, in particular in China, where the population is largest of any single nation. In addition, China has 43% of global coal production, and coal ash through combustion can produce large amount of Li, which ends up in the environment[12].

We therefore investigated the changes of [Li] and δ^7Li in the Yangtze River where it flows through two largest urban cities of Wuhan and Chongqing. This river is the world's third longest, and has most dense population and industrial development in China along its course. The population of Wuhan and Chongqing are estimated to be ~14 and 31 million, respectively, according to government statistics in 2018, higher than the Li-polluted Seoul (~12 million), South Korea[11]. We expect to observe large increase of [Li] when the Yangtze River flows across Wuhan or Chongqing, since there is 6 times increase of [Li] in the Han River crossing Seoul[11]. However, almost no variations of both [Li] and δ^7Li are observed when the Yangtze River crosses Wuhan (in a lowland region at the middle to lower reaches of the Yangtze River) during August 2006 (Supplementary Table 2). The same result is observed when the river crosses Chongqing (upper to middle reaches of the Yangtze River).

In temporal terms, we further investigated the river water collected during August 2018 around these cities, and they also show no increase of [Li] before and after draining each city (Supplementary Table 2). Also, [Li] did not increase from 2006 to 2018 in the Yangtze River.

Besides, the anthropogenic sources of Li have both higher[13-15] or lower δ^7Li values[11] relative to river water, so anthropogenic inputs may not simply result in a decrease of river δ^7Li

as the Han River.

We note that there is a case study showing Li pollution from hospital and industrial areas in a small catchment within the Loire River basin[13]. However, the spatial Li isotopes in both the tributaries and mainstem of the entire Loire basin show systematically higher values in low flow stage and lower values in high flow stage[13]. This pattern is consistent with our global observations.

Finally, for the time-series of δ^7Li in the two Tibetan catchments (Fig. 1), and the seasonal datasets both in the Pamirs streams and the headwaters of the Ganges draining the Himalayas (Fig. 2), these remote areas have obviously not been affected by human activities due to their sparse populations. These observations suggest that human activity may affect river Li levels, yet seem not be a major factor resulting in the consistent seasonal variations of δ^7Li in rivers observed across latitudes.

Supplementary Table 2. Li contents and isotopes of Yangtze draining the two largest urban cities.

River	Year	Large city	Date (mm/yy)	Sample	Site note	Li (nmol/L)	δ^7Li (‰)
Yangtze	2006	Wuhan[15]	Aug-06	CJ48	Before Wuhan	744	17.9
			Aug-06	CJ52	After Wuhan	709	17.8
		Chongqing[15]	Aug-06	CJ10	Before Chongqing	1420	12.4
			Aug-06	CJ13	After Chongqing	1470	12.6
	2018	Wuhan[16]	Aug-18	CJ17	At Wuhan	655	n.a.
			Aug-18	CJ18	After Wuhan	665	n.a.
		Chongqing[16]	Aug-18	C09	Before Chongqing	1158	n.a.
			Aug-18	C10	At Chongqing	759	n.a.

Supplementary Figures:

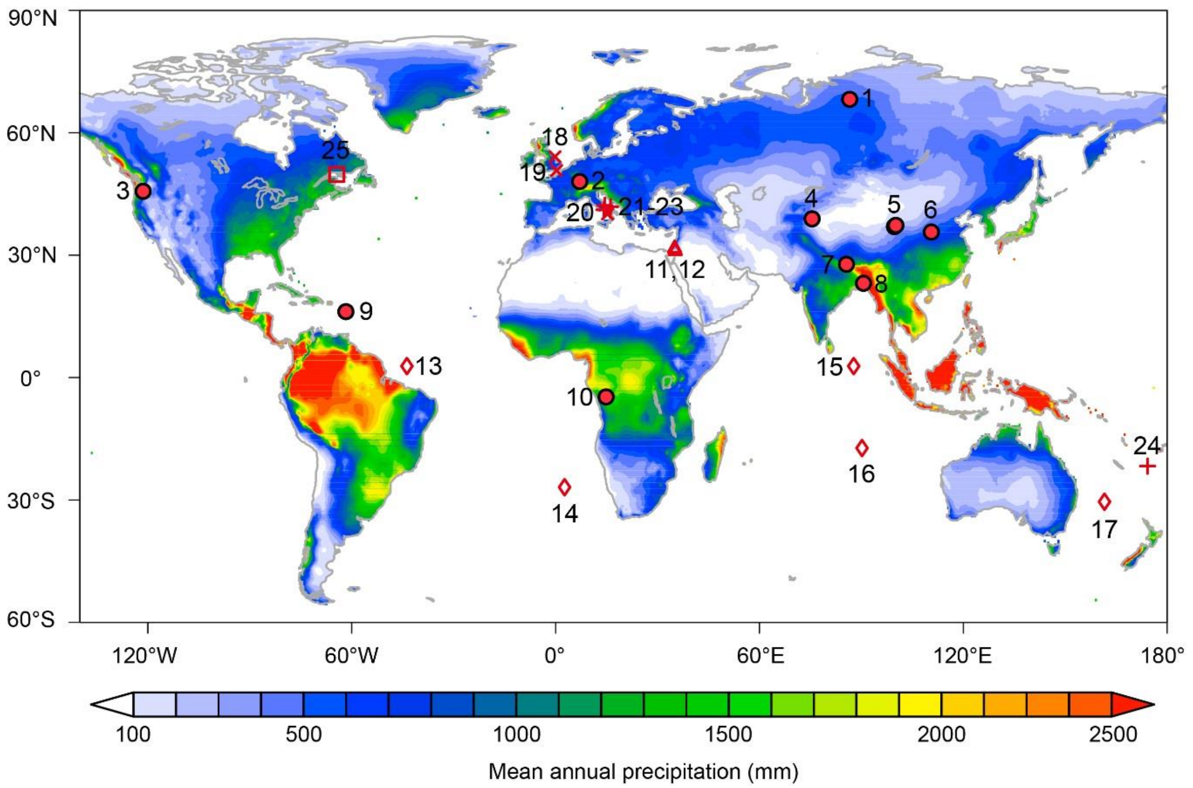

Supplementary Fig. 1 | Map of global precipitation and study areas. The mean annual precipitation during 2001-2017 is obtained from monthly climate observations from meteorological stations over the global land surface[17]. This database is constructed by Climate Research Unit of the University of East Anglia (CRU) with a 0.5°×0.5° (latitude×longitude) spatial resolution. The new and compiled study areas include: modern seasonal rivers (marked 1-10, cycles), sample locations with sample timescales across glacial cycles (11-12, triangles), Cenozoic (13-17, diamonds), Ocean Anoxic Events OAE1a (18-20, crosses) and OAE2 (21-24, multipliers), and the Hirnantian glaciation (25, squares). The map is generated by Hui Sun using the Grid Analysis and Display System (GrADS)[18] version 1.9 (Open source software).

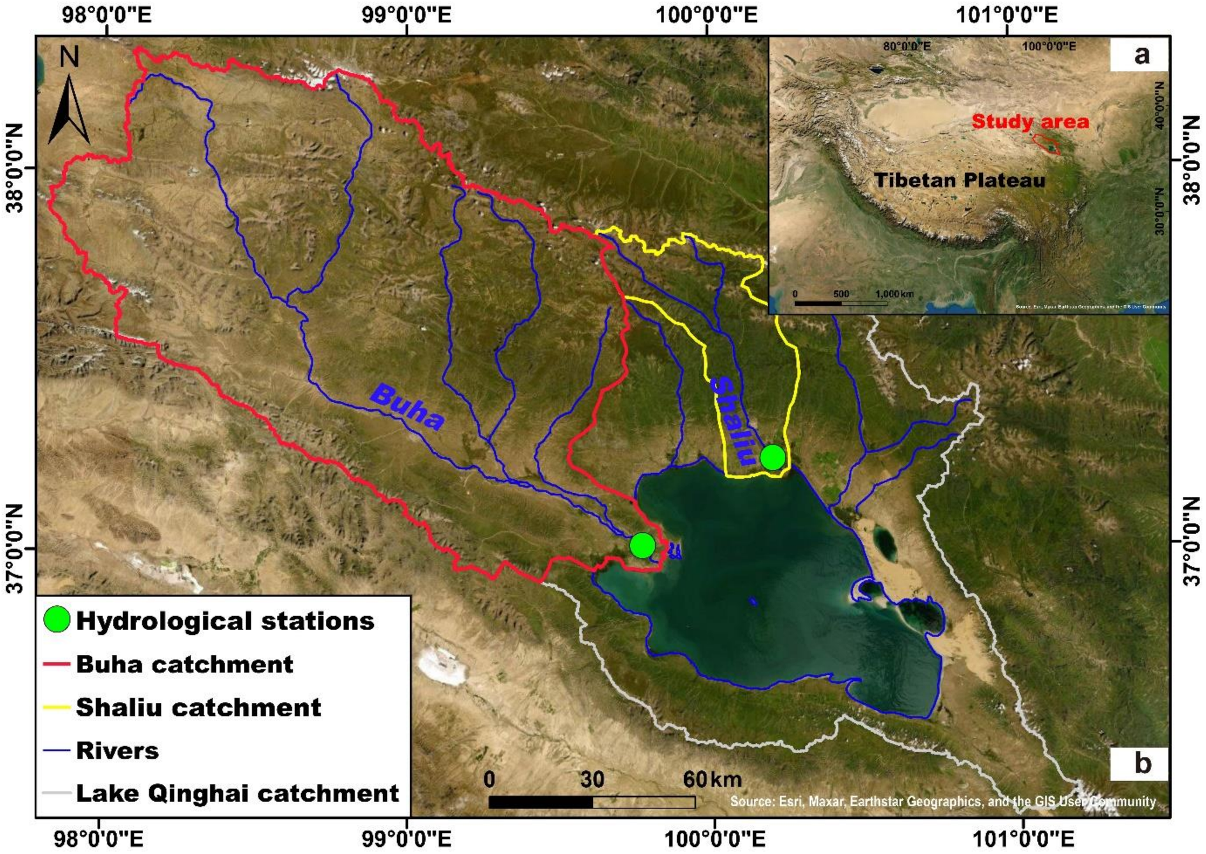

Supplementary Fig. 2 | Map of the weekly sampling sites at the northeastern Tibetan Plateau. **a** Study area in the regional context of the Tibetan Plateau. **b** Weekly sampling sites at two hydrological stations (green cycles) within the carbonate dominated Buha (BH, red-outlined polygon) and silicate dominated Shaliu (SL, yellow-outlined polygon) catchments, respectively. Background image is from ArcGIS - World Imagery (https://www.arcgis.com/home/item.html?id=10df2279f9684e4a9f6a7f08febac2a9).

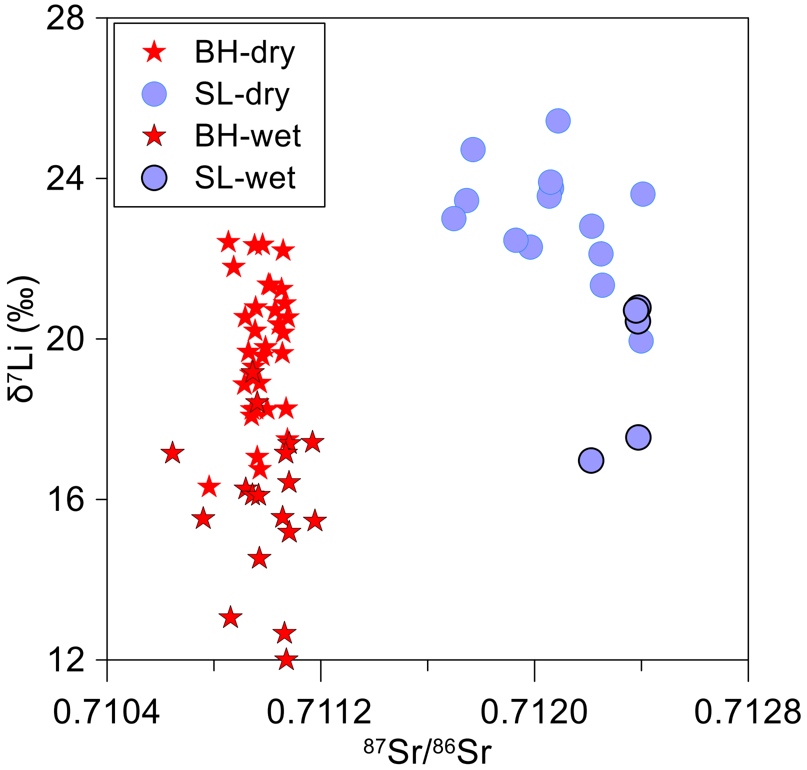

Supplementary Fig. 3 | Riverine $^{87}Sr/^{86}Sr$ versus δ^7Li at the BH and SL catchments, NE Tibetan Plateau. The overall higher $^{87}Sr/^{86}Sr$ ratios of seasonal river waters in the SL relative to the BH are consistent with the reported $^{87}Sr/^{86}Sr$ ranges of the bedrocks (BH: 0.707706-0.713996, SL: 0.711893-0.728903)[19]. There is no correlation between the $^{87}Sr/^{86}Sr$ and δ^7Li in each catchment, implying distinct control processes for the riverine Sr and Li isotopes.

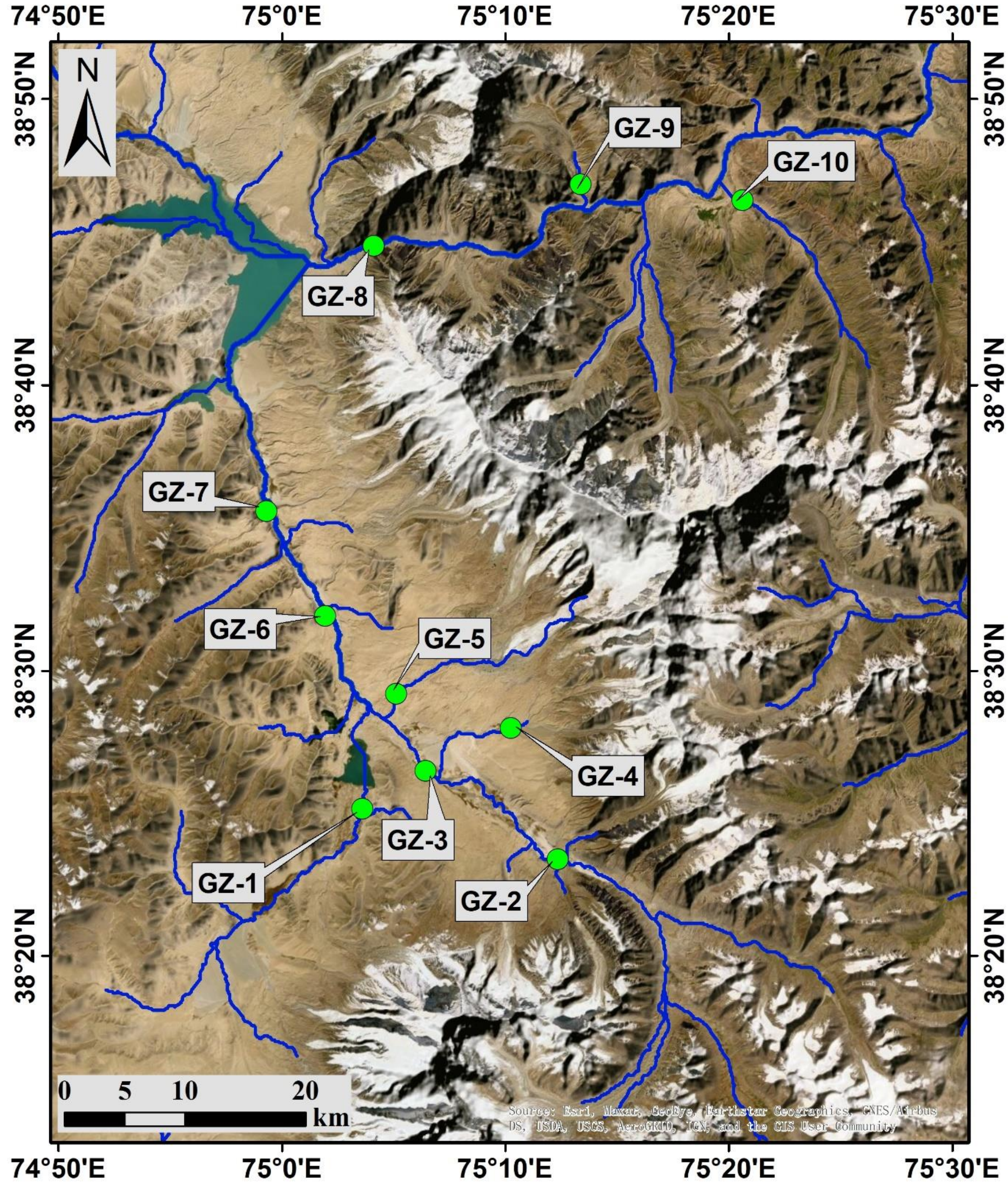

Supplementary Fig. 4 | Map of the seasonal sampling sites at the Gaizi River glacial catchment, northeastern Pamir Plateau. All sampling sites (green cycles) were collected both in late April (low ice-melting) and early September (high ice-melting) (Supplementary Data 1). Highest δ^7Li (22.9‰) occurs in a swale area at site GZ-1, reflecting a long rock-water interaction time. Background image is from ArcGIS - World Imagery (https://www.arcgis.com/home/item.html?id=10df2279f9684e4a9f6a7f08febac2a9).

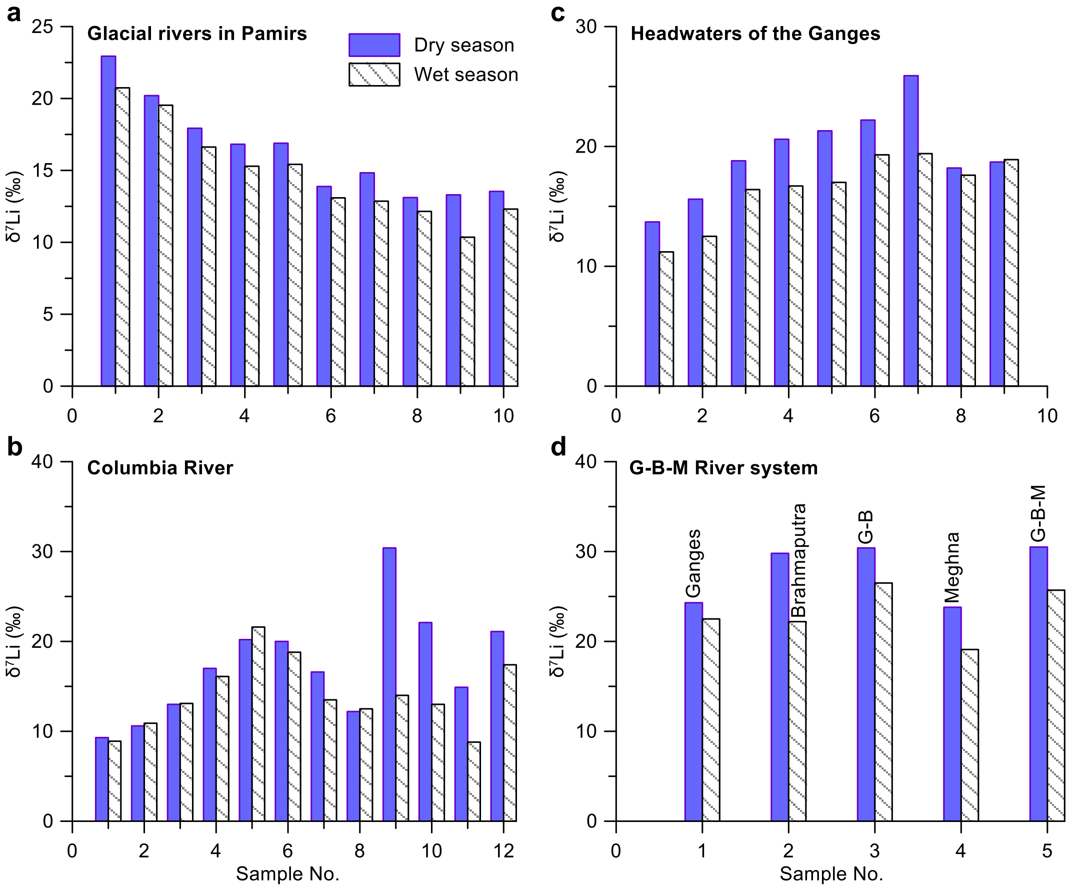

Supplementary Fig. 5 | Seasonal δ^7Li variations of spatial spot samples in different river basins. **a** Gaizi (glacial) river in the Pamir Plateau (this study). **b** Columbia River[3]. **c** Headwaters of the Ganges[5]. **d** Downstream of the Ganges-Brahmaputra-Meghna River system (G-B-M)[6]. See Supplementary Note 1 and Data 1 for details.

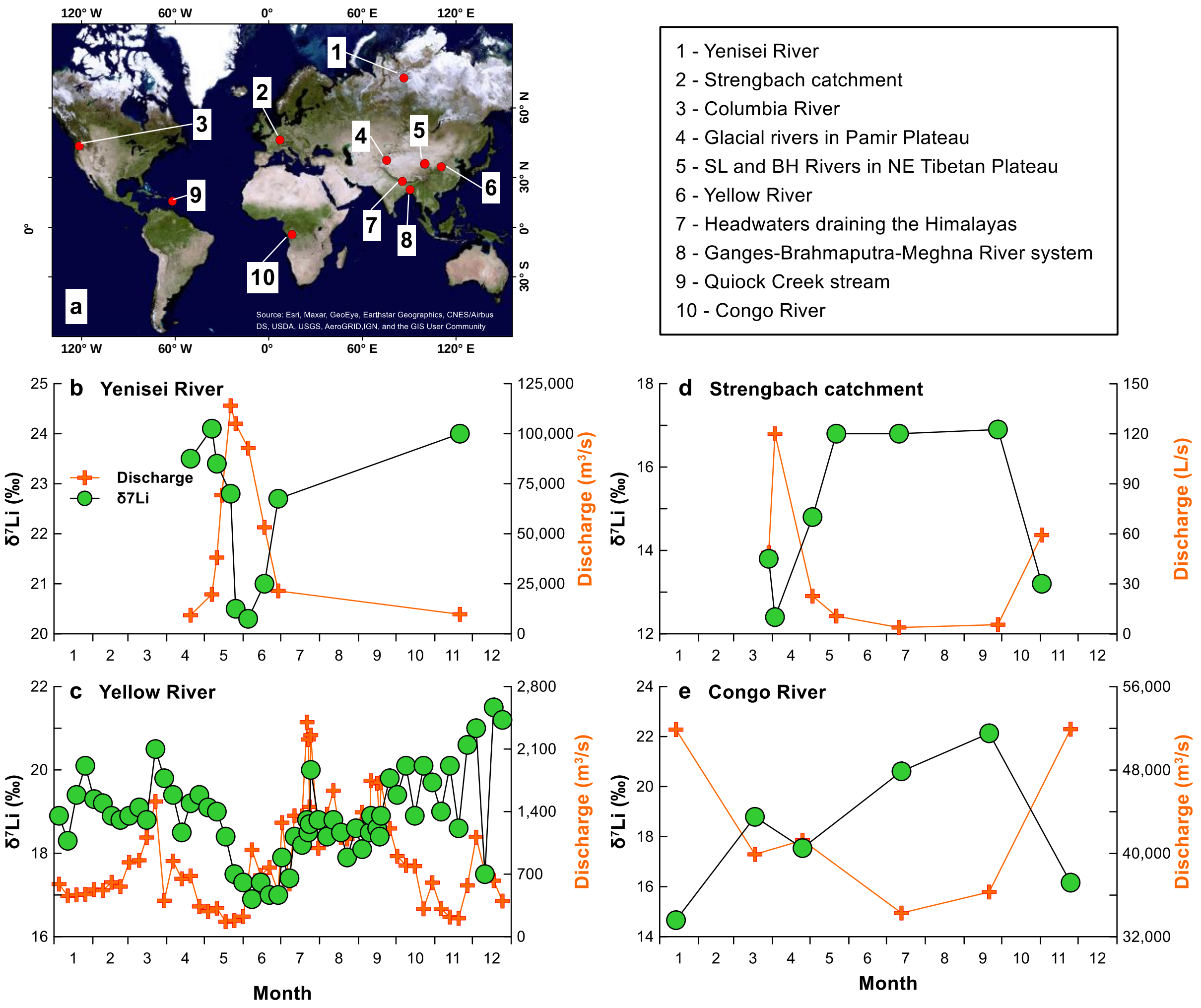

Supplementary Fig. 6 | Seasonal variations of riverine δ^{7}Li from the Arctic to the equator. **a** Map of study areas (red dots). Among the river basins, new high-resolution (weekly) variations of Li isotopes in the Buha (BH) and Shaliu (SL) catchments are shown in Fig. 1 (this study). **b-e** Compiled time-series variations of Li isotopes, showing an inverse relationship between river water discharge (orange crosses) and δ^{7}Li (green dots), consistent with the BH and SL. See Supplementary Note 1 and Data 1 for additional details and data sources. Background image (**a**) is from ArcGIS - World Imagery (https://www.arcgis.com/home/item.html?id=10df2279f9684e4a9f6a7f08febac2a9).

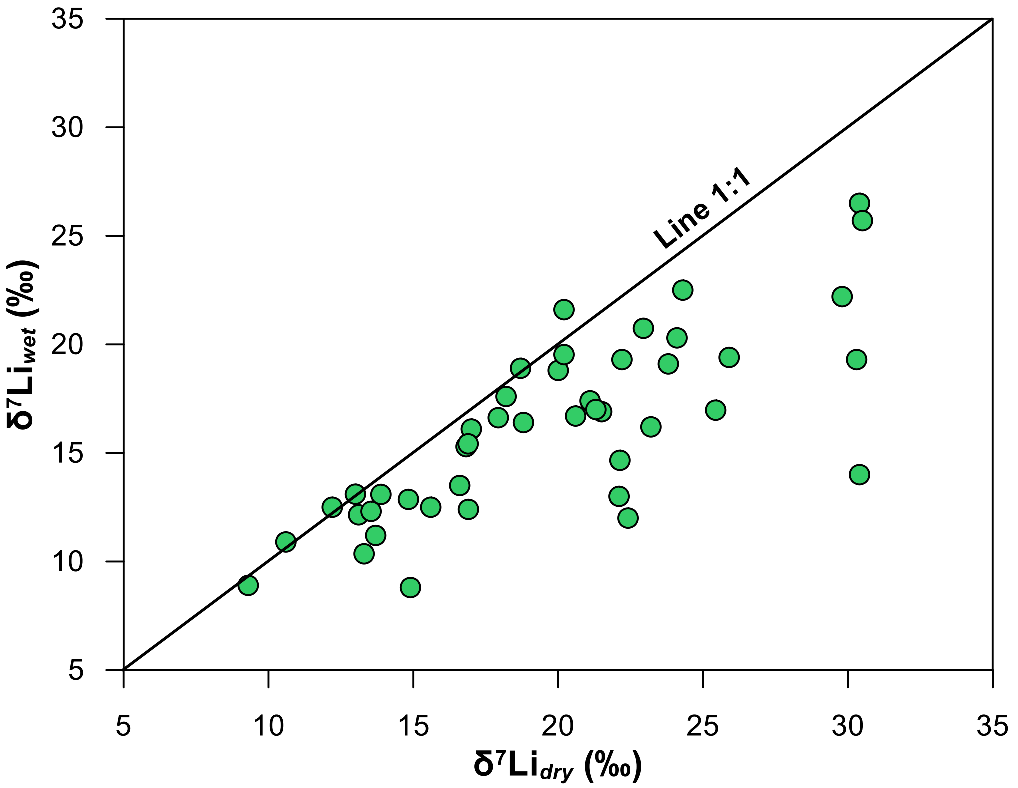

Supplementary Fig. 7 | Differences of riverine δ^{7}Li between dry and wet seasons from the Arctic to the equator. The data showing δ^{7}Li are lower in wet seasons, with a few samples having analytical errors of < ±1‰.

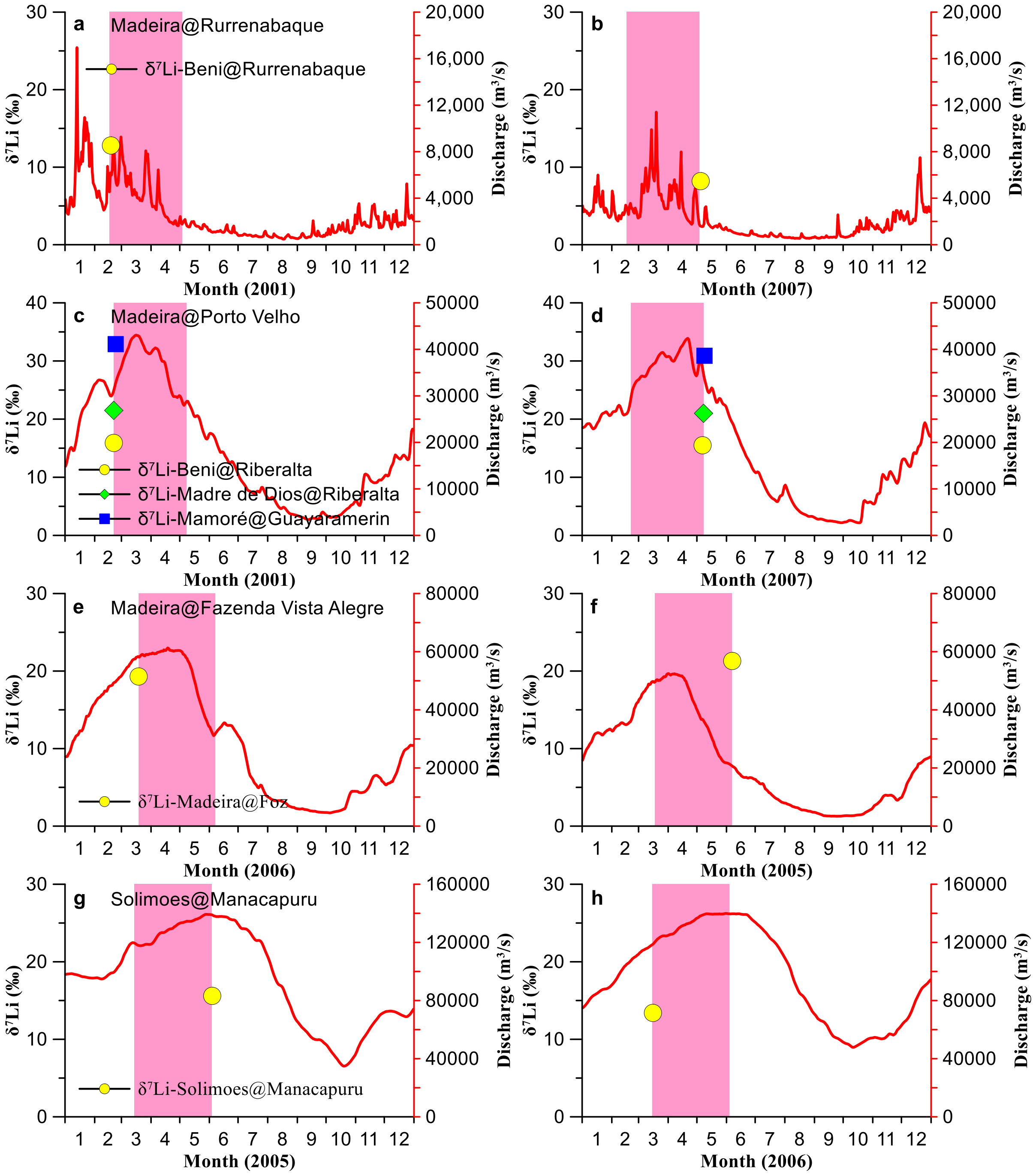

Supplementary Fig. 8 | Seasonal δ^{7}Li of river water samples from the upstream to middle section of the Amazon River. a-b The upstream of the Madeira River draining the Andes. **c-d** The Madeira River from the Riberalta to the Guayaramerin draining the flood plain. **e-f** The Madeira River mouth. **g-h** The Manacapuru of the Solimoes river draining the flood plain. These rivers show similar δ^{7}Li due to the close sampling seasons and thus similar hydrology. The daily river water discharge is monitored by the HyBAm (Hydrology of the Amazon Basin, http://www.ore-hybam.org). The δ^{7}Li data are sourced from ref.[20].

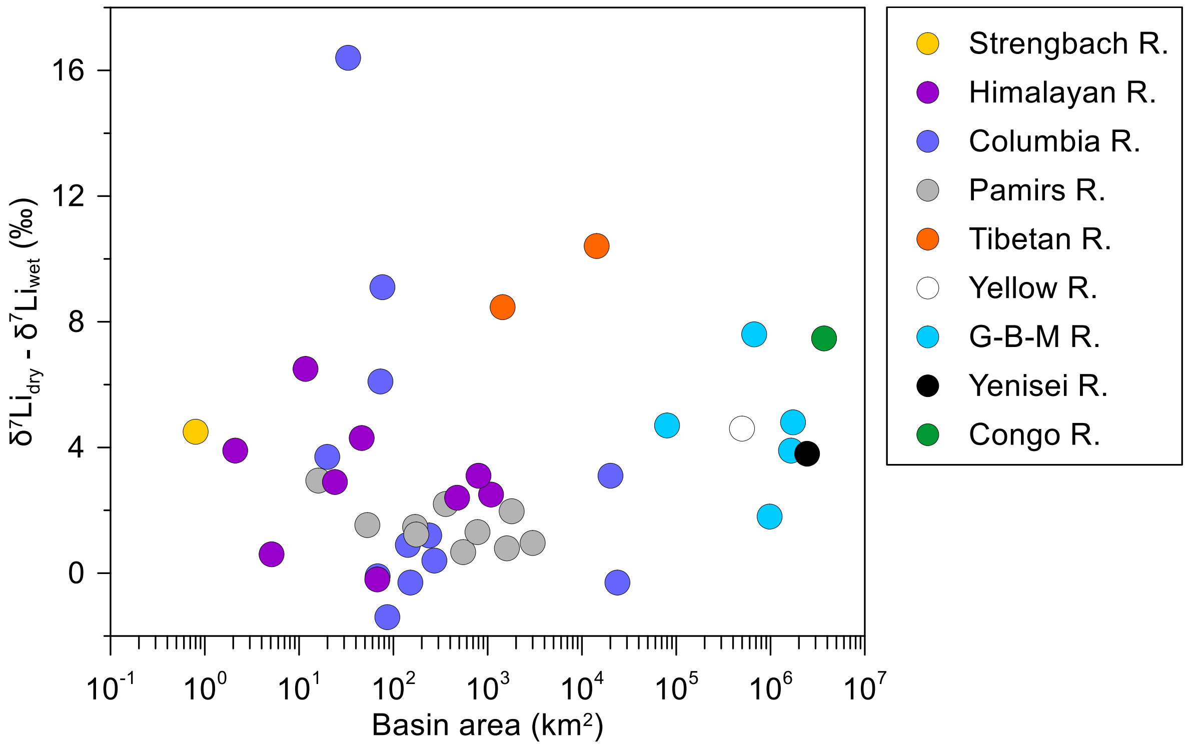

Supplementary Fig. 9 | Differences of seasonal δ^7Li changing with basin areas. (δ^7Li_{dry} - δ^7Li_{wet}) is the difference of δ^7Li between dry and wet seasons (Supplementary Data 1). The data shows that both large basins and small catchments enable to produce large seasonal δ^7Li variations, indicating that basin areas (e.g., the Yenisei, the Ganges-Brahmaputra-Meghna (G-B-M), and the Congo rivers) have no direct effects on seasonal variations.

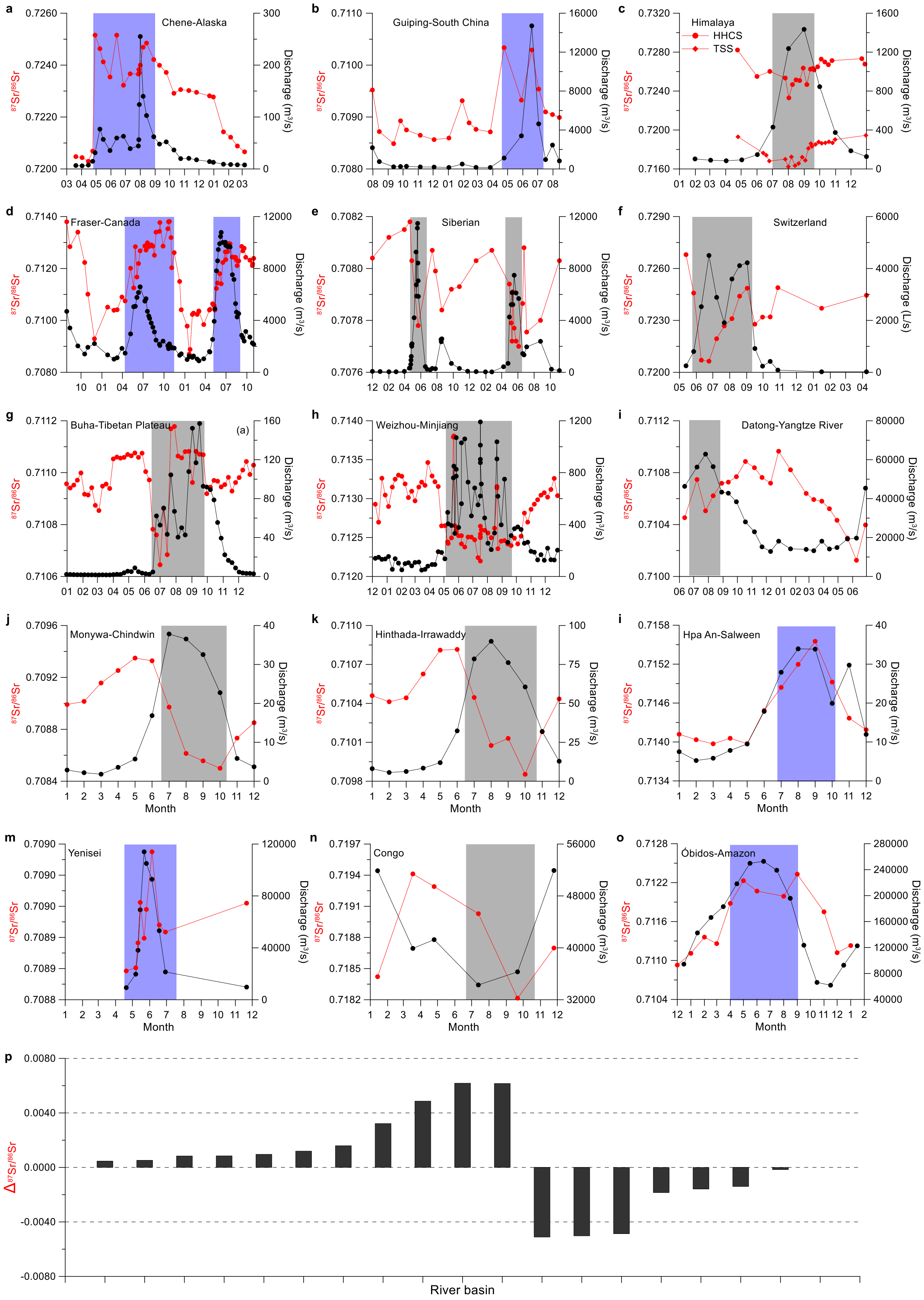

Supplementary Fig. 10 | Globally seasonal Sr isotopic variations. **a-b**, **d**, **i**, **m** and **o** showing positive correlations (purple bars) between seasonal $^{87}Sr/^{86}Sr$ (red cycles) and river water

discharges (black cycles) in the Chena River in interior Alaska[21], the Xijiang River (main channel of the Pearl River) at Guiping, south China[22], the Fraser River in Canada[22], the Salween River at Hpa-An[24], the Yenisei River draining to the Arctic[1], and the Amazon River at Óbidos[25], while others showing inverse covariations (grey bars) between them, including the Himalayan headwaters in the Tethyan Sedimentary Series (TSS) and the High Himalayan Crystalline Series (HHCS)[26], the Siberian Kochechum River draining permafrost dominated areas[27], the glacial stream in Switzerland[28], the Buha River in the Tibetan Plateau[29], the Minjiang River at Weizhou, a major tributary of the Yangtze River[30], the downstream Yangtze River at Datong[31], the Chindwin River at Monywa[24], Irrawaddy River at Hinthada[24], and the Congo River at Kinshasa[7]. **p** Differences of riverine $^{87}Sr/^{86}Sr$ ratios from dry to wet seasons in the above river basins (Supplementary Data 3). Both inverse and covariation relationships reflect complex control processes, such as lithology control. In **c** both Sr data from HHCS and TSS areas are shown.

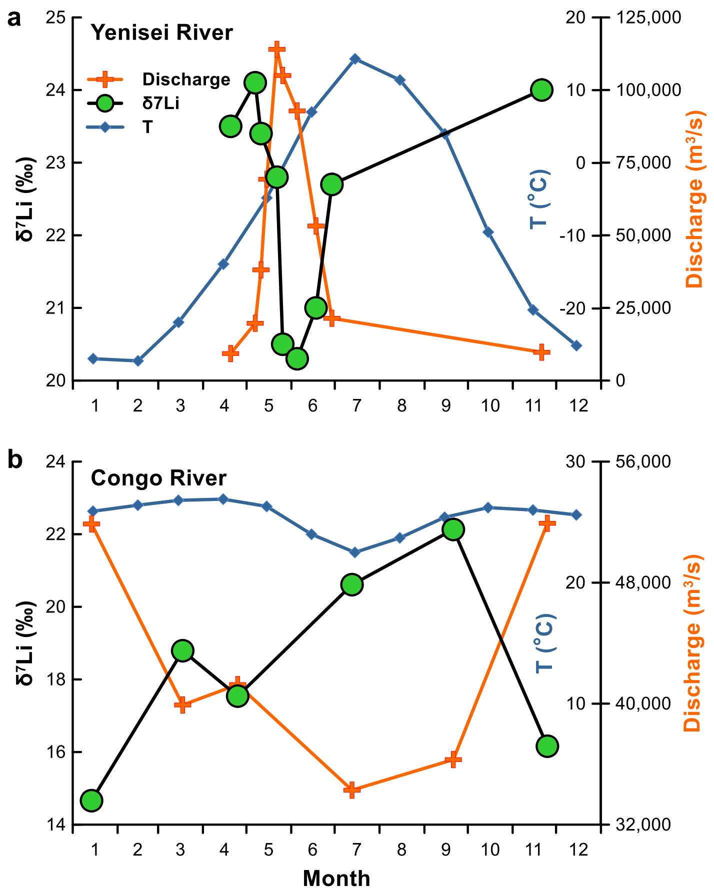

Supplementary Fig. 11 | The relationship between air temperature and seasonal δ^{7}Li. a The seasonal air temperature (T, blue squares) in the Yenisei River shows an offset with both the river water discharge (orange crosses) and δ^{7}Li (green dots). The river water discharge in this arctic river reaches the peak during spring melting season, but not yet for the temperature. **b** The air temperature almost stays constant with negligible seasonality, excluding the major temperature control on δ^{7}Li variations in the Congo rivers. Both rivers show a clear hydrology dependence of Li isotopes. Temperature data in Yenisei River (**a**) at Norilsk is from refs.[32-34], and in Congo (**b**) at Kinshasa from ref.[35].

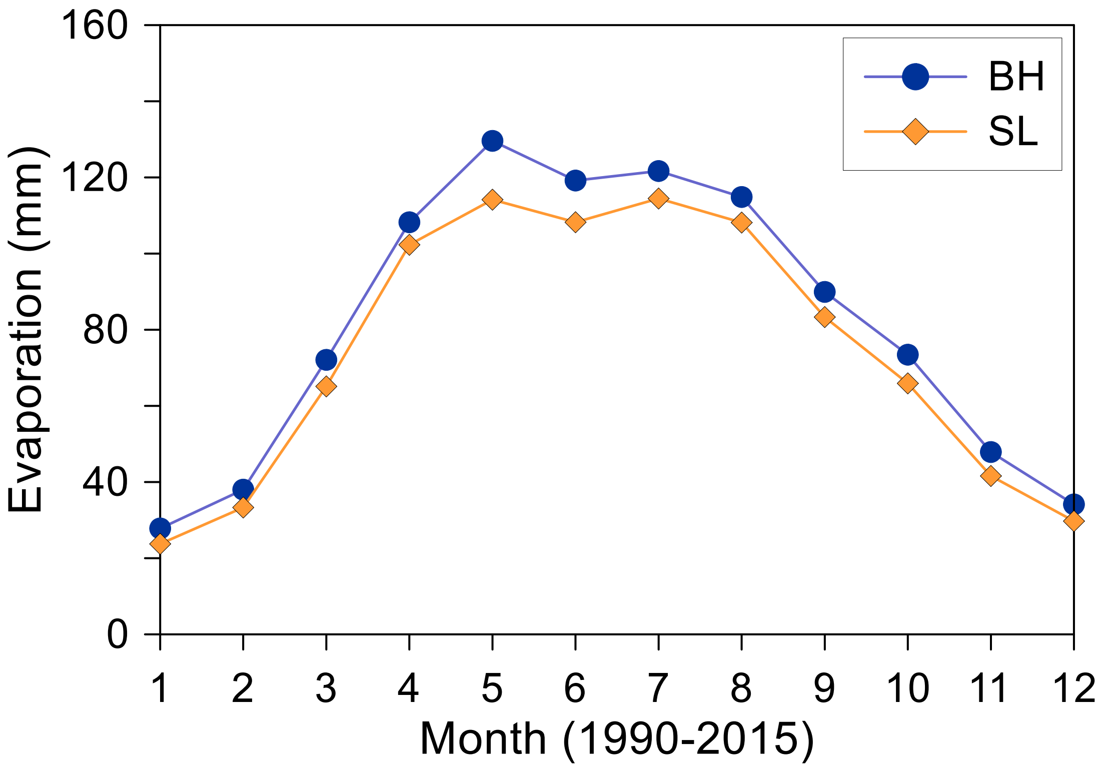

Supplementary Fig. 12 | Monthly evaporation within the BH and SL catchments. The data indicates much higher evaporation during wet season in both Buha (BH) and Shaliu (SL) catchments. The monthly values are averages during 1990 to 2015. The data are monitored by local hydrological stations within each catchment.

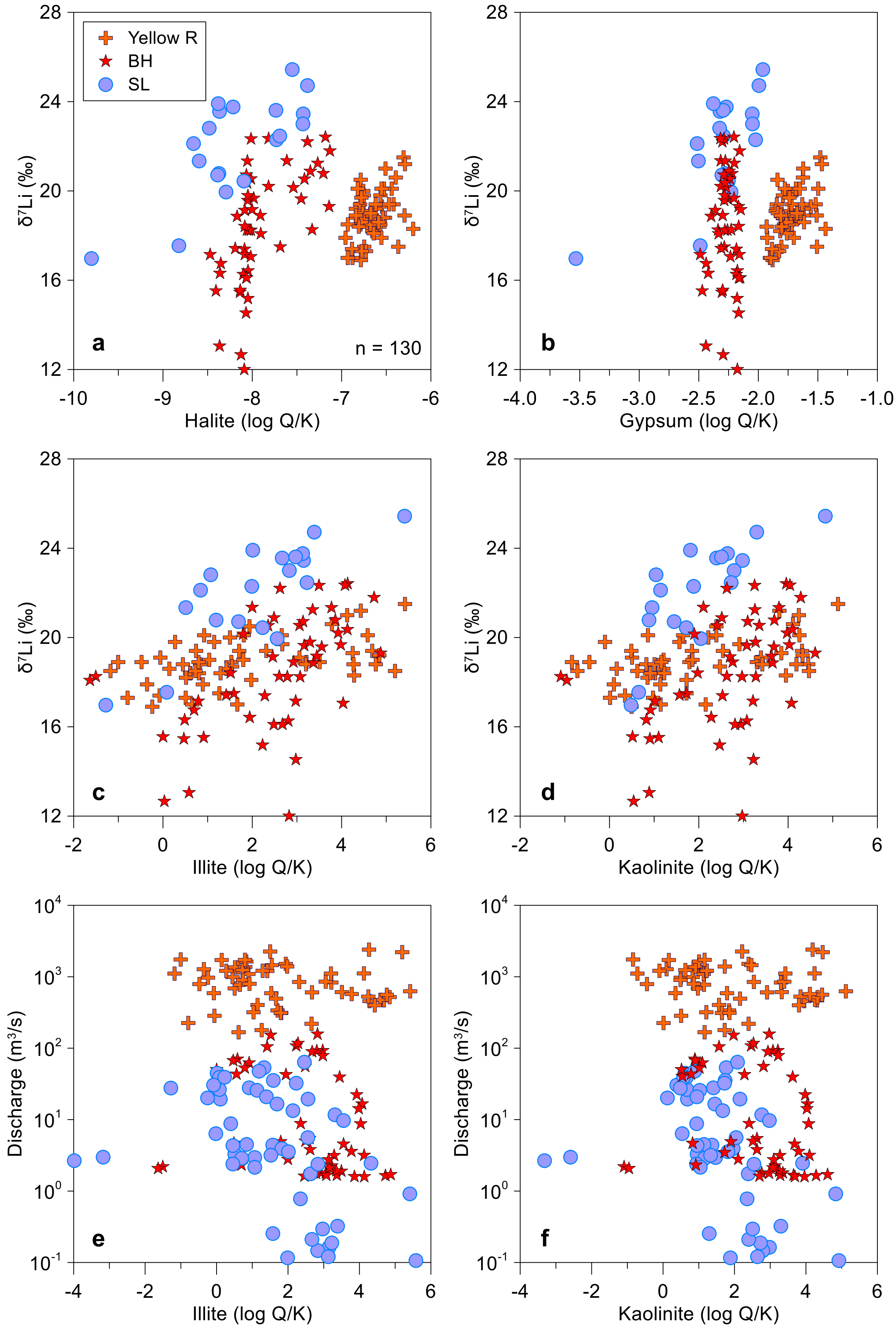

Supplementary Fig. 13 | The relationships between seasonal δ^7Li and saturation index of minerals in the BH, SL and Yellow Rivers. The riverine δ^7Li values show no correlations with saturation states of evaporites (**a-b**), but weak positive correlations with clay minerals (**c-d**). The data imply that decreased runoff with longer residence times enhances saturation state of secondary clay minerals (**e-f**), resulting in higher river δ^7Li. The mineral saturation indexes were calculated using Geochemist's Workbench V.8.0.

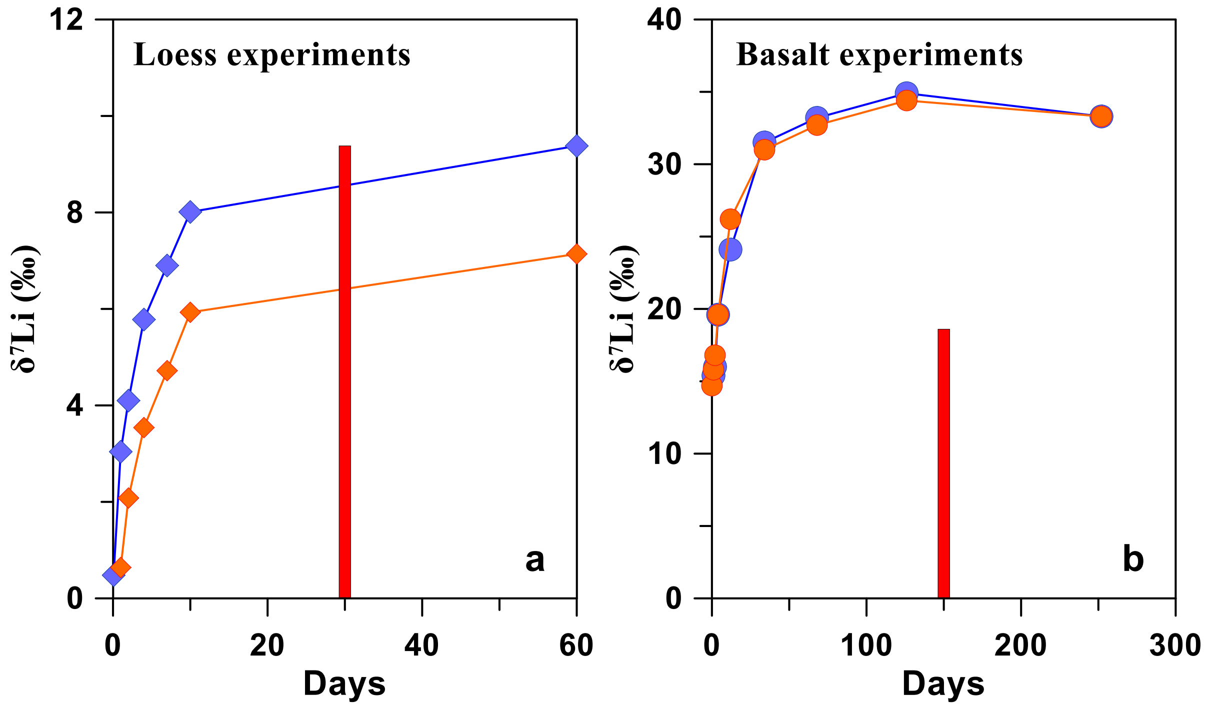

Supplementary Fig. 14 | Dissolution experiments of loess and basalt. **a**, Dissolution experiments of loess in ultrapure water[36] showed that aqueous δ^7Li increased by 7.38‰ and 9.14‰ within 60 days with loess contents of 16.4 g/L (orange) and 32.8 g/L (blue), respectively. **b**, Laboratory experiments of water-rock interaction[37] found ~19‰ increase of δ^7Li by reacting basalt sand with natural river water over 9 months (orange and blue). Their results indicated that removal of Li via secondary mineral formation, together with rapidly sorption of Li onto secondary phases, largely affected the Li isotopic fractionations. Both experiments, showing increasing δ^7Li with residence times (**a-b**), provide robust evidence that residence times could cause rapid (days to months) and remarkable Li isotopic fractionations. Red bars are the differences of dissolved δ^7Li from the start to the end time of the experiments.

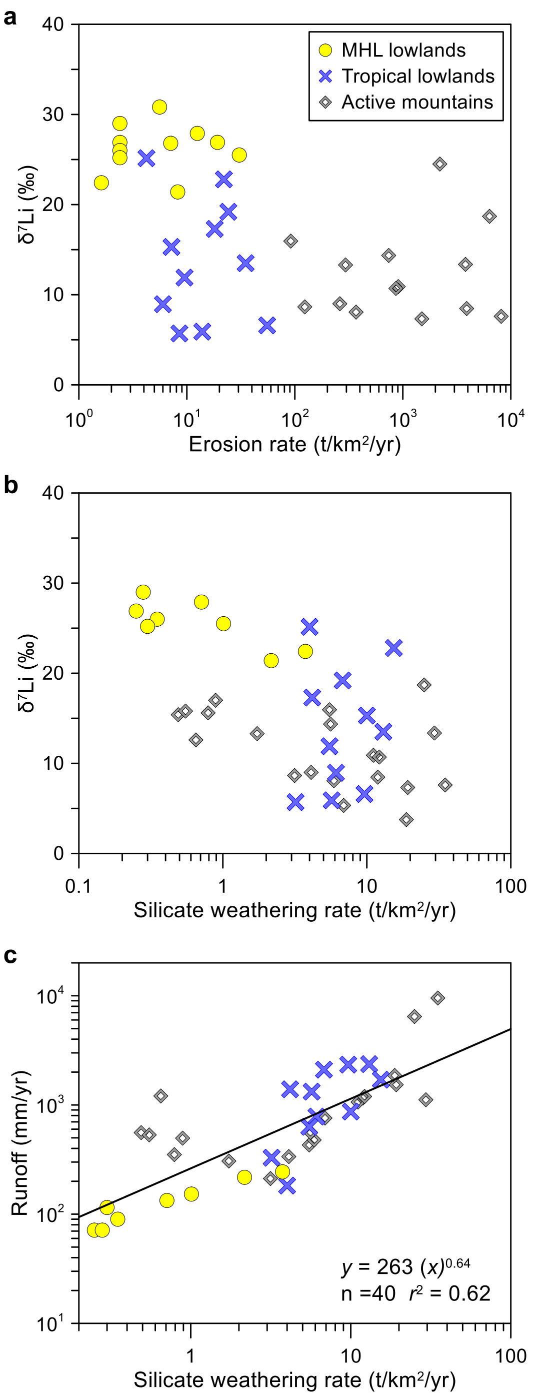

Supplementary Fig. 15 | The relationships between riverine δ^{7}Li and rates of weathering and erosion. **a** δ^{7}Li versus erosion rates. **b** δ^{7}Li versus silicate weathering rates. **c** Runoff versus silicate weathering rates. **a** and **b** show that the middle-high latitude (MHL) lowlands have highest δ^{7}Li but lowest silicate weathering and erosion rates, while active mountains have overall lower δ^{7}Li but highest weathering and erosion rates (Supplementary Data 2). In addition, although tropical lowlands have similar δ^{7}Li and runoff with uplifted mountains (Fig. 3b), their erosion rates are lower, indicating that mountain uplift produces higher erosion rate but not necessarily higher ^{7}Li-riched river waters. The positive relationship in **c** shows that although active mountains and lowlands have distinct tectonic backgrounds, their silicate weathering rates still follow a runoff dependence.

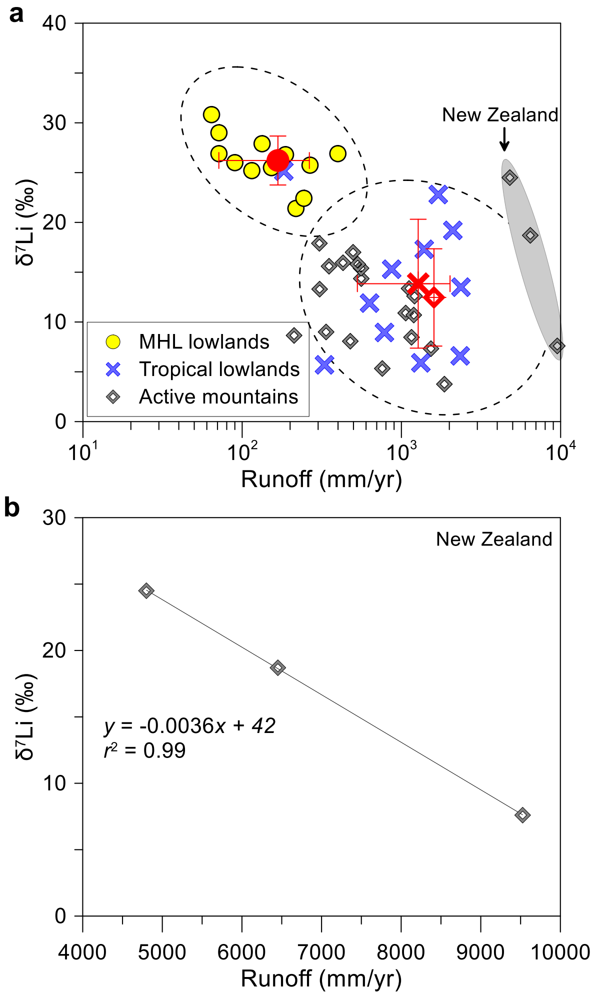

Supplementary Fig. 16 | The relationships between riverine δ⁷Li and runoff. **a** Lowlands and active mountain areas. **b** New Zealand. When amplifying the 3 outliers (grey ellipse) of the New Zealand in **a**, there is still a strong runoff control of Li isotopes, showing ~20‰ decline of δ^7Li as runoff increase from ~4500 to ~9500 mm/yr (**b**). The overall deviation of the 3 New Zealand data implies that in addition to a major hydrology control, there are other factors that likely contribute to the riverine δ^7Li. See Fig. 3 for legend details.

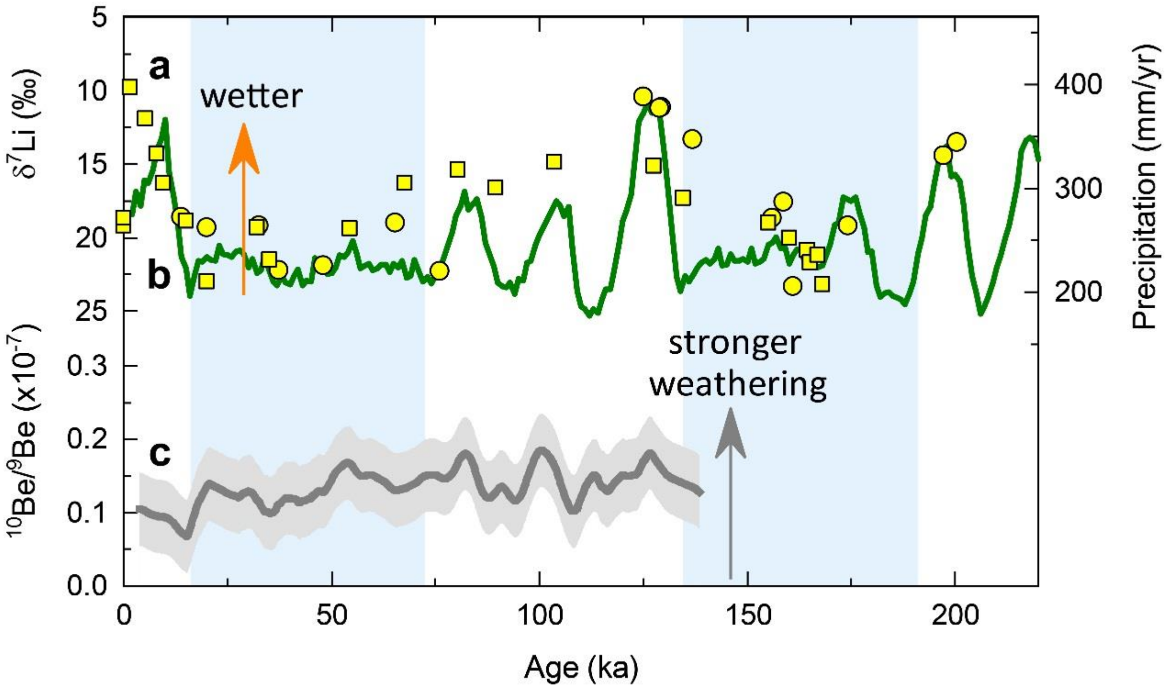

Supplementary Fig. 17 | Glacial δ^7Li, ^{10}Be/^{9}Be records and climate. **a** Mediterranean speleothems δ^7Li (yellow) from two Israeli caves during the last glacial cycle[38]. **b** Simulated annual mean precipitation (mm/yr, 1000-year average) (light green) of the Lake Ohrid in the Mediterranean region[39]. **c** Corrected ^{10}Be/^{9}Be records (grey solid line) of Mediterranean outflow water[40]. In glacial period (**a**), δ^7Li tightly linked to hydrologic changes, rather than to continental weathering (**a**-**c**). Errors for δ^7Li are similar to the symbol size; different symbols represent samples are different locations. Shadings in **c** show a maximum uncertainty (10%) for the ^{10}Be/^{9}Be correction. Vertical light blue bars indicate relatively dry intervals.

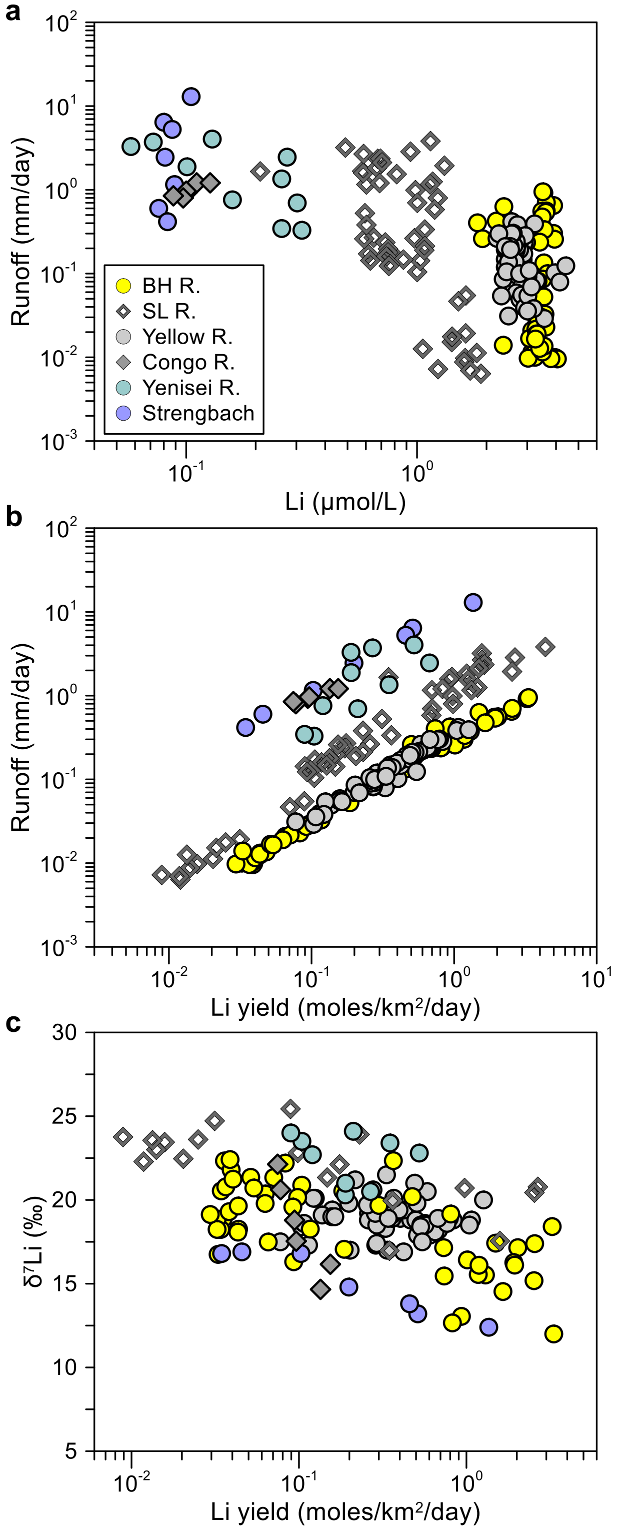

Supplementary Fig. 18 | Runoff control on riverine Li concentrations, Li yield and δ^{7}Li for the time-series dataset across latitudes. a Runoff versus Li concentrations. **b** Runoff versus Li yield. **c** δ^{7}Li versus Li yield. These time-series data indicate that high runoff dilutes river water Li concentrations, but still results in high Li yield and coupled with low δ^{7}Li. This observation is also consistent with the spatial results in Fig. 3. The BH and SL mean Buha and Shaliu rivers.

Supplementary References:

1 Hindshaw, R. S., Teisserenc, R., Le Dantec, T. & Tananaev, N. Seasonal change of geochemical sources and processes in the Yenisei River: A Sr, Mg and Li isotope study. *Geochimica et Cosmochimica Acta* **255**, 222-236, doi:10.1016/j.gca.2019.04.015 (2019).

2 Lemarchand, E., Chabaux, F., Vigier, N., Millot, R. & Pierret, M.-C. Lithium isotope systematics in a forested granitic catchment (Strengbach, Vosges Mountains, France). *Geochimica et Cosmochimica Acta* **74**, 4612-4628, doi:10.1016/j.gca.2010.04.057 (2010).

3 Liu, X.-M., Wanner, C., Rudnick, R. L. & McDonough, W. F. Processes controlling δ7Li in rivers illuminated by study of streams and groundwaters draining basalts. *Earth and Planetary Science Letters* **409**, 212-224, doi:10.1016/j.epsl.2014.10.032 (2015).

4 Gou, L.-F. *et al.* Li isotopes in the middle Yellow River: Seasonal variability, sources and fractionation. *Geochimica et Cosmochimica Acta* **248**, 88-108, doi:10.1016/j.gca.2019.01.007 (2019).

5 Kısakűrek, B., James, R. H. & Harris, N. B. W. Li and δ7Li in Himalayan rivers: Proxies for silicate weathering? *Earth and Planetary Science Letters* **237**, 387-401, doi:10.1016/j.epsl.2005.07.019 (2005).

6 Manaka, T. *et al.* Downstream and seasonal changes of lithium isotope ratios in the Ganges-Brahmaputra river system. *Geochemistry, Geophysics, Geosystems* **18**, 3003-3015, doi:10.1002/2016gc006738 (2017).

7 Henchiri, S., Gaillardet, J., Dellinger, M., Bouchez, J. & Spencer, R. G. M. Riverine dissolved lithium isotopic signatures in low-relief central Africa and their link to weathering regimes. *Geophysical Research Letters* **43**, 4391-4399, doi:10.1002/2016gl067711 (2016).

8 Fries, D. M. Temporal variations in continental weathering processes: Insights from Li and Mg isotopes, *University of Southampton, Doctoral Thesis*, (2018).

9 Misra, S. & Froelich, P. N. Lithium isotope history of Cenozoic seawater: changes in silicate weathering and reverse weathering. *Science* **335**, 818-823, doi:10.1126/science.1214697 (2012).

10 Pogge von Strandmann, P. A. E., Jenkyns, H. C. & Woodfine, R. G. Lithium isotope evidence for enhanced weathering during Oceanic Anoxic Event 2. *Nature Geoscience* **6**, 668-672, doi:10.1038/ngeo1875 (2013).

11 Choi, H. B., Ryu, J. S., Shin, W. J. & Vigier, N. The impact of anthropogenic inputs on lithium content in river and tap water. *Nat Commun* **10**, 5371, doi:10.1038/s41467-019-13376-y (2019).

12 Schlesinger, W. H., Klein, E. M., Wang, Z. & Vengosh, A. Global Biogeochemical Cycle of Lithium. *Global Biogeochemical Cycles*, doi:10.1029/2021gb006999 (2021).

13 Millot, R. & Négrel, P. Lithium isotopes in the Loire River Basin (France): Hydrogeochemical characterizations at two complementary scales. *Applied Geochemistry* **125**, doi:10.1016/j.apgeochem.2020.104831 (2021).

14 Négrel, P., Millot, R., Petelet-Giraud, E. & Klaver, G. Li and δ7Li as proxies for weathering and anthropogenic activities: Application to the Dommel River (meuse basin). *Applied Geochemistry* **120**, doi:10.1016/j.apgeochem.2020.104674 (2020).

15 Wang, Q.-L. *et al.* Behavior of lithium isotopes in the Changjiang River system: Sources effects and response to weathering and erosion. *Geochimica et Cosmochimica Acta* **151**, 117-132, doi:10.1016/j.gca.2014.12.015 (2015).

16 Xu, Y. *et al.* Carbonate weathering dominates magnesium isotopes in large rivers: Clues from the Yangtze River. *Chemical Geology* **588**, doi:10.1016/j.chemgeo.2021.120677 (2022).

17 Mitchell, T. D. & Jones, P. D. An improved method of constructing a database of monthly climate observations and associated high-resolution grids. *International Journal of Climatology* **25**, 693-712, doi:10.1002/joc.1181 (2005).
18 Center for Ocean-Land-Atmosphere Studies (COLA), Grid Analysis and Display System (GrADS). Available at: http://cola.gmu.edu/grads/gadoc/gadoc.php.
19 Jin, Z., Wang, S., Zhang, F. & Shi, Y. Weathering, Sr fluxes, and controls on water chemistry in the Lake Qinghai catchment, NE Tibetan Plateau. *Earth Surface Processes and Landforms* **35**, 1057-1070, doi:10.1002/esp.1964 (2010).
20 Dellinger, M. *et al.* Riverine Li isotope fractionation in the Amazon River basin controlled by the weathering regimes. *Geochimica et Cosmochimica Acta* **164**, 71-93, doi:10.1016/j.gca.2015.04.042 (2015).
21 Douglas, T. A., Blum, J. D., Guo, L., Keller, K. & Gleason, J. D. Hydrogeochemistry of seasonal flow regimes in the Chena River, a subarctic watershed draining discontinuous permafrost in interior Alaska (USA). *Chemical Geology* **335**, 48-62, doi:10.1016/j.chemgeo.2012.10.045 (2013).
22 Wei, G. *et al.* Seasonal changes in the radiogenic and stable strontium isotopic composition of Xijiang River water: Implications for chemical weathering. *Chemical Geology* **343**, 67-75, doi:10.1016/j.chemgeo.2013.02.004 (2013).
23 Voss, B. M. *et al.* Tracing river chemistry in space and time: Dissolved inorganic constituents of the Fraser River, Canada. *Geochimica et Cosmochimica Acta* **124**, 283-308, doi:10.1016/j.gca.2013.09.006 (2014).
24 Chapman, H., Bickle, M., Thaw, S. H. & Thiam, H. N. Chemical fluxes from time series sampling of the Irrawaddy and Salween Rivers, Myanmar. *Chemical Geology* **401**, 15-27, doi:10.1016/j.chemgeo.2015.02.012 (2015).
25 Santos, R. V. *et al.* Source area and seasonal87Sr/86Sr variations in rivers of the Amazon basin. *Hydrological Processes* **29**, 187-197, doi:https://doi.org/10.1002/hyp.10131 (2014).
26 Tipper, E. T. *et al.* The short term climatic sensitivity of carbonate and silicate weathering fluxes: Insight from seasonal variations in river chemistry. *Geochimica et Cosmochimica Acta* **70**, 2737-2754, doi:10.1016/j.gca.2006.03.005 (2006).
27 Bagard, M.-L. *et al.* Seasonal variability of element fluxes in two Central Siberian rivers draining high latitude permafrost dominated areas. *Geochimica et Cosmochimica Acta* **75**, 3335-3357, doi:10.1016/j.gca.2011.03.024 (2011).
28 Hindshaw, R. S. *et al.* Hydrological control of stream water chemistry in a glacial catchment (Damma Glacier, Switzerland). *Chemical Geology* **285**, 215-230, doi:10.1016/j.chemgeo.2011.04.012 (2011).
29 Jin, Z. *et al.* Seasonal contributions of catchment weathering and eolian dust to river water chemistry, northeastern Tibetan Plateau: Chemical and Sr isotopic constraints. *Journal of Geophysical Research* **116**, doi:10.1029/2011jf002002 (2011).
30 Jin, Z. *et al.* Seismically enhanced solute fluxes in the Yangtze River headwaters following the A.D. 2008 Wenchuan earthquake. *Geology* **44**, 47-50, doi:10.1130/g37246.1 (2016).
31 Luo, C. *et al.* Tracing Sr isotopic composition in space and time across the Yangtze River basin. *Chemical Geology* **388**, 59-70, doi:10.1016/j.chemgeo.2014.09.007 (2014).
32 Noril'sk climate. Weatherbase. Retrieved May 16 (2019).
33 Climate Noril'sk. MeteoBlue. Retrieved May 16 (2019).
34 Norilsk, Russia - Monthly weather forecast and Climate data". Weather Atlas. Retrieved May 16 (2019).
35 Climate: Kinshasa. AmbiWeb GmbH. Archived from the original on 9 May 2016. Retrieved 7 June (2016).

36 Liu, C.-Y. Behavior of lithium isotopes during loess dissolutionand its implication as a silicate weathering tracer. *Masteral dissertation, University of Chinese Academy of Sciences* (2019).
37 Pogge von Strandmann, P. A. E. *et al.* Experimental determination of Li isotope behaviour during basalt weathering. *Chemical Geology* **517**, 34-43, doi:10.1016/j.chemgeo.2019.04.020 (2019).
38 Pogge von Strandmann, P. A. E. *et al.* Lithium isotopes in speleothems: Temperature-controlled variation in silicate weathering during glacial cycles. *Earth and Planetary Science Letters* **469**, 64-74, doi:10.1016/j.epsl.2017.04.014 (2017).
39 Wagner, B. *et al.* Mediterranean winter rainfall in phase with African monsoons during the past 1.36 million years. *Nature* **573**, 256-260, doi:10.1038/s41586-019-1529-0 (2019).
40 von Blanckenburg, F., Bouchez, J., Ibarra, D. E. & Maher, K. Stable runoff and weathering fluxes into the oceans over Quaternary climate cycles. *Nature Geoscience* **8**, 538-542, doi:10.1038/ngeo2452 (2015).
