# Supplementary material for: Hydrological control of river and seawater lithium isotopes
Source: Nat Commun. 2022 Jun 10;13:3359. doi: 10.1038/s41467-022-31076-y (PMC9187753; doi:10.1038/s41467-022-31076-y)
Supplement: Supplementary file 3 — Description of Additional Supplementary Files [file 41467_2022_31076_MOESM3_ESM.pdf]

Description of Additional Supplementary Files

Supplementary Data 1: Global seasonal Li isotopes.

Supplementary Data 2: Spatial Li isotopes of lowland and mountain rivers.

Supplementary Data 3: Global seasonal Sr isotopes.
